# Screening of the key volatile organic compounds of *Tuber melanosporum* fermentation by aroma sensory evaluation combination with principle component analysis

**DOI:** 10.1038/srep17954

**Published:** 2015-12-11

**Authors:** Rui-Sang Liu, Guang-Huai Jin, Deng-Rong Xiao, Hong-Mei Li, Feng-Wu Bai, Ya-Jie Tang

**Affiliations:** 1 School of Life Science and Biotechnology, Dalian University of Technology, Dalian 116024 China; 2 Key Laboratory of Fermentation Engineering (Ministry of Education), Hubei Provincial Key Laboratory of Industrial Microbiology, Hubei University of Technology, Wuhan 430068 China

## Abstract

Aroma results from the interplay of volatile organic compounds (VOCs) and the attributes of microbial-producing aromas are significantly affected by fermentation conditions. Among the VOCs, only a few of them contribute to aroma. Thus, screening and identification of the key VOCs is critical for microbial-producing aroma. The traditional method is based on gas chromatography-olfactometry (GC-O), which is time-consuming and laborious. Considering the *Tuber melanosporum* fermentation system as an example, a new method to screen and identify the key VOCs by combining the aroma evaluation method with principle component analysis (PCA) was developed in this work. First, an aroma sensory evaluation method was developed to screen 34 potential favorite aroma samples from 504 fermentation samples. Second, PCA was employed to screen nine common key VOCs from these 34 samples. Third, seven key VOCs were identified by the traditional method. Finally, all of the seven key VOCs identified by the traditional method were also identified, along with four others, by the new strategy. These results indicate the reliability of the new method and demonstrate it to be a viable alternative to the traditional method.

*Tuber melanosporum*, commonly known as the “black diamond of cuisine”, is highly appreciated for its unique and characteristic aroma^1^,^2^,^3^. Fruiting-body of *Tuber melanosporum* on the market comes mainly from natural or semi-artificial cultivation. However, the former is scarce, and the latter is time-consuming: cultivation usually takes 4–12 years to harvest the fruiting-body^4^,^5^. A submerged fermentation technique for *Tuber* production of mycelia and its metabolites was first developed in our lab as a promising alternative method for its fruiting-body^6^,^7^,^8^,^9^.

Aroma is one of the important characteristics of the *T*. *melanosporum* fermentation system. Aroma results from the interplay of volatile organic compounds (VOCs), and more than 200 VOCs have been identified from natural *Tuber* fruiting-bodies^10^,^11^,^12^,^13^,^14^. During submerged fermentation of *T. melanosporum*, the formation of aroma attributes not only was affected by the interplay of VOCs, but was also significantly affected by fermentation conditions. In our previous study, 59 VOCs were identified in the *T. melanosporum* submerged fermentation system^15^, and the fermentation conditions had greater impact than the truffle species on the profile of VOCs^16^. Among the fermentation conditions, the absence of a major carbon source of sucrose and the shift of culture temperature had the most significant effects on the profile of VOCs, followed by the absence of yeast extract or peptone^17^. Among these VOCs reported from the *Tuber* fruiting-bodies, the volatile organic sulfur-containing compounds (VOSCs) such as methanethiol, dimethyl sulfide, dimethyl disulfide, dimethyl trisulfide, 3-(methylthio)-propanal, and 3-(methylthio)-1-propanol, were also first detected in *T. melanosporum* submerged fermentation by the addition of 5 g/L L-methionine^18^. These results indicated that it was possible to adjust the VOC profile by control of fermentation conditions.

It has been shown that the overall VOCs-profile contains a large number of VOCs. Accordingly, only certain VOCs are key contributors to aroma^19^. Therefore, the screening and identification of the key VOCs plays an important role during an aroma production process. The traditional method, composed of the following three steps, is typically used. First, the VOCs extracted from each fermentation sample are quantitatively analyzed by gas chromatography-mass spectrometry (GC-MS). Second, the flavor dilution (FD) factor for each VOC is determined by aroma extract dilution analysis (AEDA). The samples are often diluted in increasing dilution order (i.e., 1:2, 1:4, 1:8, 1:16, etc.) and are then evaluated by the sniffers^19^. The flavor dilution (FD) factor for a particular VOC is determined by identifying the greatest dilution level that still provides detectable aroma. For example, if a diluted sample of 1:16 could be detected by sniffers but could not be detected at a 1:32 dilution, the FD would then be 16. Third, the odor activity value (OAV) is used to study the contribution of each key VOC to the original aroma. The OAV of individual compounds is calculated as the ratio of its concentration in the fermentation samples to its odor threshold^20^. It is well-accepted that an individual VOC with a higher FD factor will have a greater contribution to aroma, and a higher OAV indicates a more active role in the aroma attributes^21^,^22^,^23^. However, this traditional method is based on gas chromatography-olfactometry (GC-O) technology, which is time-consuming and laborious for screening and identifying the key VOCs during production of microbial-producing aromas. The considerable labor and time needed for screening could be due to two main reasons. The first is that we do not know exactly what types of aroma attributes are favorable, and the second is that using olfactory sense to screen the key VOCs for aroma from many fermentation samples is very challenging.

To address this problem, taking the *T. melanosporum* fermentation system as a typical example, a new strategy, presented herein, was developed to screen and identify the key VOCs for aroma by combining the aroma evaluation method with principle component analysis (PCA). More precisely, this work included (1) an aroma sensory evaluation method to screen the favorite aroma fermentation samples, (2) PCA was employed to screen and identify the key VOCs from the favorite aroma sample, (3) the traditional method was also employed to screen and identify the key VOCs from the favorite aroma sample and (4) the reliability of the new strategy was verified by comparing the key VOCs identified from the new strategy and the traditional method.

## Results

### The development of aroma sensory evaluation method

Considering the smell of fermentation medium without inoculation as the control, the smell of the control was set at a score of 30 as the base line (Table 1). When *T. melanosporum* was inoculated in a fermentation medium, an alcohol smell gradually began to appear during the fermentation process. According to the intensity of the alcohol smell, the alcohol smell can be divided into varying degrees of detectability: faint (when alcohol is just barely detectable), moderate (when the smell is obvious but not over-powering) and strong (when the smell is strong). The score of faint, moderate, and strong alcohol was set from 1 to 5, from 6 to 10, and from 11 to 20, respectively. Once the presence of alcohol in the fermentation sample reached strong, the score of the fermentation system was the base line (30) plus from 11 to 20, depending on the sensory intensity of the panelist.

In addition to the above basic aroma characteristics, four other types of attributes (i.e., sulfurous^10^, earthy^24^, mushroom^25^, and green^26^ were important for *Tuber* fruiting-bodies. As a promising alternative for *Tuber* fruiting-bodies, these four types of aroma attributes were also expected to be discernible in the *Tuber* fermentation system. Thus, an additional score was developed. As shown in Table 1, each aroma attribute of sulfurous, mushroom, earthy, green, and others (fruity, flowery, sweet, hay) was marked from 0 to 10 and was once again dependent on the sensory intensity of the panelist. A score of zero means that the aroma characteristic is absent; a score of 10 means that the aroma attribute is strongly present but does not completely overwhelm the basic aroma attribute. So, if one fermentation sample’s score was larger than 50, its aroma attribute of alcohol should coexist with one or more additional aroma characteristics. Therefore, a total additional score could range from 0 to 50 by the addition of the score of each term (mushroom, flowery, sulfurous, green, and others).

To conclude, adding the base score to the additional score yields the sensory aroma score. A fermentation sample with a sensory aroma score larger than 50 was necessary to be considered an aroma-producing sample.

### Aroma-producing fermentation condition optimization

As shown in Table 2, when a 5, 10, or 15 g/L yeast extract was combined with 35 g/L of glucose, the scores of fermentation samples were all less than 50. When the glucose concentration was increased from 35 to 60 g/L, the scores were significantly enhanced and 13 sample scores were larger than 50, ranging from 51.5 ± 1.6 to 61.4 ± 0.8. Furthermore, when the glucose concentration was increased to 80 g/L, the scores were not significantly enhanced and 11 sample scores were larger than 50, ranging from 52.1 ± 1.8 to 59.0 ± 1.0. Among the sets, five samples (from day 3 to day 7) had scores larger than 50 for G60Y15 (i.e., 60 g/L glucose and 15 g/L yeast extract) indicating that the G60Y15 was favorable for aroma production. However, when the glucose was replaced by sucrose, there were only five samples with scores larger than 50, and the largest score of 52.5 ± 1.5 was obtained on day 7 of S80Y10 (i.e., 80 g/L sucrose and 10 g/L yeast extract). Thus, sucrose was not suitable for aroma production.

The effect of culture temperature (25, 28, 30, and 32 °C) on the sensory aroma scores was subsequently investigated (Table 2). All of the scores obtained at the culture temperature of 32 °C were less than the medium smell base line (i.e., score = 30). There were five, four, and four sample scores larger than 50 under the culture temperature of 25, 28, and 30 °C, respectively, and the highest score for each temperature was 61.4 ± 0.8 (day 6), 71.6 ± 2.1 (day 6), and 55.0 ± 9.4 (day 6), respectively. Clearly, the culture temperature of 28 °C, which provided the highest score, is most suitable for aroma production.

Based on the above optimal case (i.e., G60Y15, 28 °C), the effect of culture pH on the sensory aroma scores was studied. There were five sample scores larger than 50 for a case where the initial pH was set to 7.0 and then left uncontrolled, and there were one, zero, and three scores larger than 50 for pH levels maintained at 6.0, 5.5, and 4.0, respectively. The highest score of 71.6 ± 2.1 (day 6), and 68.5 ± 9.9 (day 7) was obtained at initial pH was 7.0 then uncontrolled and pH controlled stability at 4.0, respectively. While the pH controlled stability at 5.5 leading all of the samples’ scores were less than 41. Therefore, initial pH of 7.0 then without control pH was suitable for aroma production.

To conclude, the optimal fermentation conditions (hereafter referred to as the control for the following studies) for the aroma-producing by *T. melanosporum* fermentation was 60 g/L glucose combined with 15 g/L yeast extract maintained at a temperature of 28 °C with an uncontrolled pH that was initialized to 7.0. The highest sensory aroma score of 71.6 ± 2.1 and the most satisfying aroma attributes from the fermentation sample were obtained from these optimal fermentation conditions.

### Quantitative analysis of VOCs and principle component analysis

The types and concentrations of VOCs were significantly affected by the fermentation conditions. Thus, qualitative and quantitative analysis of the VOC production was studied. As shown in Table 3, a total of 29 VOCs were identified from the fermentation samples (Table 2). These VOCs were composed of a series of compounds including alcohols, pyrazines, aldehydes, ketones, esters, aromatic compounds, and oximes. Meanwhile, the relative amounts (calculated by relative peak area) of the 29 VOCs were also calculated. As previously mentioned, sensory aroma scores larger than 50 were considered to be indicative of sufficient aroma-producing samples. There were 40 fermentation samples (enumerated in Table 2) with sensory aroma scores larger than 50. Among the 40 fermentation samples, six samples were not considered to have favorite aroma attributes, including five samples with scores ranging from 50.5 ± 2.9 to 52.5 ± 1.5 that were obtained using sucrose as the carbon source, and one sample with a score of 50.0 ± 11.7 that had a culture pH of 6.0. Thus, the remaining 34 samples obtained from nine fermentation conditions (i.e., G80Y5, G80Y10, G80Y15, G60Y5, G60Y10, G60Y15, T30, pH 4.0, Control) were studied in the following process.

**Figure 1:**
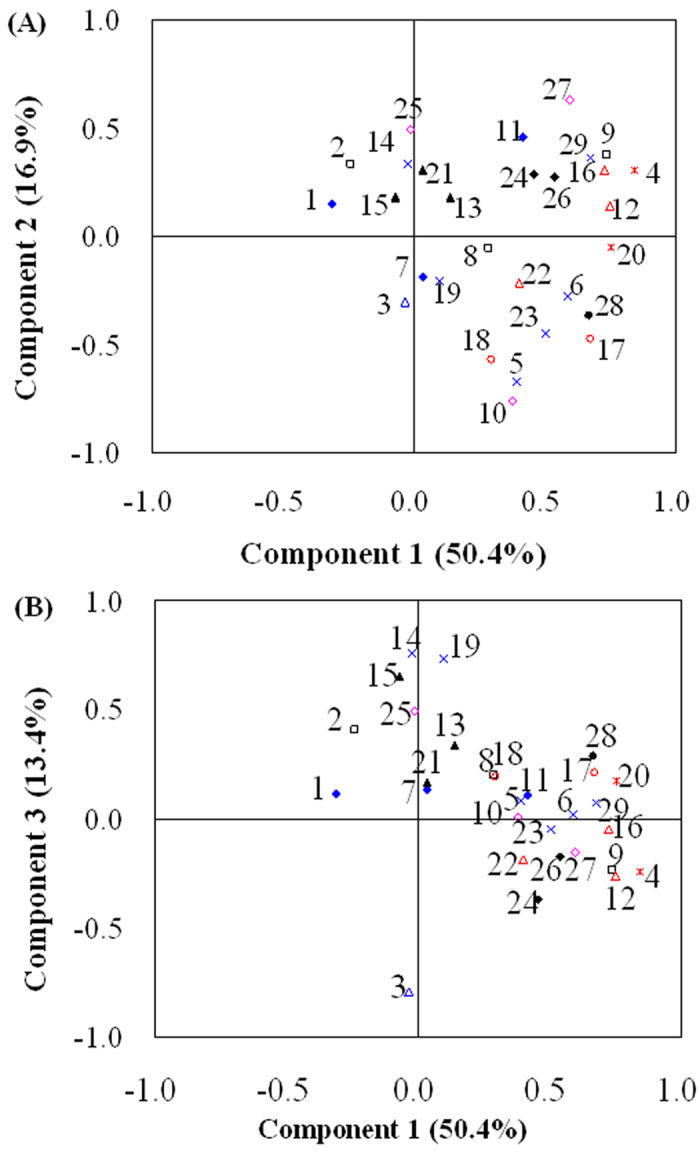
The three first principle components of 29 VOCs from the 34 fermentation samples of nine conditions with sensory aroma scores larger than 50. Score plots of PC_1_ − PC_2_ (**A**) and PC_1_ − PC_3_ (**B**) were shown. The number that follows refers to the compounds of Table 3.

A matrix of data composed of relative peak areas of 29 VOCs calculated from the 34 fermentation samples were analyzed using PCA. To summarize the procedure, PCA was used to identify the VOCs that had the most distinguishable attributes. This analysis was limited to the first three principle components (PCs) of the PCAs because the cumulative variance analysis of the first principle component (PC_1_) to the third principle component (PC_3_) accounted for 80.7%, a percentage sufficiently high to ensure that the PCA plots were representative of the main features of the data set. As can be observed in Fig. 1A (PC_1_ VS. PC_2_), 2,3-butanediol (NO. 4), 3-(methylthio)-1-propanol (No. 9), benzeneacetaldehyde (NO. 12), 4-ethylphenol (NO. 16), and 2,5-dimethyl-3-propylpyrazine (NO. 20) can be found in the upper-left quadrant with an absolute value of contribution larger than 0.65 to the PC_1_, indicating that they were most important to the PC_1_. 2,5-dimethylpyrazine (NO. 5), and trimethylpyrazine (NO. 10) can be found in the lower-left quadrant with an absolute value of contribution larger than 0.65 to the PC_2_, indicating that they were most important to the PC_2_. As shown in Fig. 1B (PC_1_ VS. PC_3_), the 3-methyl-1-butanol (NO. 3) can be found centered-below, with an absolute value of contribution larger than 0.65 to the PC_3_, while the 3-ethyl-2,5-dimethylpyrazine (NO. 14), phenylethyl alcohol (NO. 15), and 3-butyl-2,5-dimethylpyrazine (NO. 19) can be found centered-above with an absolute value of contribution larger than 0.65 to the PC_3_, indicating these VOCs were most important to the PC_3_. As summarized above in Table 4, a total above 11 VOCs were identified as key VOCs to the favorite aroma.

The formation of aroma was significantly affected by the types and concentrations of VOCs. Thus, the effects of the content of the 11 key VOCs providing the favorite aroma attributes were studied. First, the average relative peak areas was calculated by the total relative peak area for each of these 11 key VOCs obtained under nine different fermentation conditions (i.e., G80Y5, G80Y10, G80Y15, G60Y5, G60Y10, G60Y15, T30, pH 4.0, and control) with sensory aroma scores larger than 50. Second, these values of average relative peak area and the sensory aroma scores of the control (i.e., the optimal fermentation conditions for the aroma produced by *T. melanosporum*) were considered as the standards for comparison. Specifically, (1) if the relative peak area for a key VOC for a particular fermentation condition were larger than the average relative peak area, while its sensory aroma score were lower than that of the control, this would indicate that a lower VOC concentration for this fermentation condition would be favorable in terms of aroma; (2) for a key VOC for a different fermentation condition, a relative peak area larger than the average relative peak area and a sensory aroma score similar to that of the control indicates that the VOC concentration for this fermentation condition is not important to the aroma; (3) a relative peak area lower than the average relative peak area and a sensory aroma score lower than that of the control indicates that a higher VOC concentration would be favorable; (4) a relative peak area lower than the average relative peak area with a sensory aroma score similar to that of the control indicates that this VOC concentration would be not important; (5) a relative peak area similar to the average relative peak area and a sensory aroma score also similar to that of the control indicates that this VOC and its concentration would be very important.

As shown in Table 5, when the relative peak areas of 4-ethylphenol (NO. 16) and 3-propyl-2,5-dimethyl-3-pyrazine (NO. 20) were small, there were no significance in sensory aroma scores for the fermentation conditions of G80Y5, G80Y10, G80Y15, G60Y5, G60Y10, G60Y15, and T30. These results showed that these VOCs (NO. 16 and 20) were not the key VOCs in the favorite aroma. The relative peak areas of VOCs were compared to the average relative peak area for the control (65.05 ± 6.75) and for samples with higher sensory aroma scores (Table 5), obtained under the fermentation condition of pH 4.0 (62.17 ± 6.97, calculated as the average sensory aroma score of the samples with sensory aroma scores larger than 50). It was observed that both the relative peak areas of 3-methyl-1-butanol (NO. 3) and phenylethyl alcohol (NO. 15) were quite similar to the average relative peak area indicating that 3-methyl-1-butanol and phenylethyl alcohol were key VOCs in the favorite aroma. The relative peak areas of the control and 2,3-butanediol (NO. 4) obtained under the fermentation condition of pH 4.0 were all larger than the average relative peak area, showing that a higher relative peak area of these VOCs were beneficial to the favorite aroma. The relative peak areas of 3-ethyl-2,5-dimethylpyrazine (NO. 14) obtained under the fermentation condition of pH 4.0 and the control were 0.24 and 0.31, respectively, which are both lower than the average relative peak area (0.42), indicating that a lower level of 3-ethyl-2,5-dimethylpyrazine was beneficial to the favorite aroma. In addition, the concentrations of the other five VOCs (i.e., 2,5-dimethylpyrazine (NO. 5), 3-(methylthio)-1-propanol (NO. 9), trimethylpyrazine (NO. 10), benzeneacetaldehyde (NO. 12), and 3-butyl-2, 5-dimethylpyrazine (NO. 19)) were not considered as key VOCs for the favorite aroma. This was concluded as the relative peak areas of these five VOCs were either larger or lower than that of the average relative peak area, and the sensory aroma scores obtained for the pH 4.0 condition and the control were not significantly different.

The relative peak areas of 2,3-butanediol (NO. 4), 3-(methylthio)-1-propanol (NO. 9), benzeneacetaldehyde (NO. 12) obtained under the fermentation conditions of G80Y5, G80Y10, G80Y15 were all lower than that of the average relative peak area, showing that a higher relative peak area of these VOCs would be beneficial to the favorite aroma. The relative peak areas of 3-(methylthio)-1-propanol (NO. 9), benzeneacetaldehyde (NO. 12), and 3-ethyl-2, 5-dimethylpyrazine (NO. 14) obtained under the fermentation conditions of G60Y5, G60Y10, G60Y15 were all lower than that of the average relative peak area, showing that a higher relative peak area of these VOCs would be beneficial to the favorite aroma.

Compared with the relative peak areas calculated from the fermentation condition of T30, the control (i.e., T28), and the average relative peak area, it was easily found that higher levels of 3-methyl-1-butanol (NO. 3) and trimethylpyrazine (No. 10) and lower level of phenylethyl alcohol (NO. 15) would be beneficial to the favorite aroma. In addition, the relative peak areas of 2, 5-dimethylpyrazine (NO. 5), benzeneacetaldehyde (NO. 13), 3-butyl-2, 5-dimethylpyrazine (NO. 19) were quite similar to the average relative peak area, showing their importance to the favorite aroma.

To conclude, 9 VOCs, i.e., 3-methyl-1-butanol (NO. 3), 2,3-butanediol (NO. 4), 2,5-dimethylpyrazine (NO. 5), 3-(methylthio)-1-propanol (NO. 9), trimethylpyrazine (No. 10), benzeneacetaldehyde (NO. 12), 3-ethyl-2, 5-dimethylpyrazine (NO. 14), phenylethyl alcohol (NO. 15), and 3-butyl-2,5-dimethylpyrazine (NO. 19), were identified as the key VOCs for the favorite aroma.

### Verification test

To verify the results obtained above, the traditional method, i.e., using aroma extract dilution analysis (AEDA) and OAV, was also used to identify the key VOCs to the desired aroma attributes from the culture time of day 2 to day 7 under the optimal aroma-producing fermentation conditions. As shown in Table 6, seven compounds were found in the AEDA within the range of 1 to 32. The most important compounds having the highest FD factors within the range of 16 to 32 were 2, 5-dimethylpyrazine (NO. 5) and 3-butyl-2, 5-dimethylpyrazine (NO. 19), which exhibited nutty and raw potato, and green odor qualities, respectively. The second most important group of compounds included 3-methyl-1-butanol (NO. 3-cheese characteristics), 3-ethyl-2, 5-dimethylpyrazine (NO. 14-nutty and roasted-potato), phenylethyl alcohol (NO. 15-rose flower odor quality). The third most important group of compounds included 3-(methylthio)-1-propanol (NO. 9) and benzeneacetaldehyde (NO. 12) with the highest FD factor was 1. These seven VOCs were identified to be key contributors to the aroma.

The OAV listed in Table 6 was also used to determine the relative contribution of each key VOC to the aroma. 3-(methylthio)-1-propanal (No. 9) had the highest OAV of 117, followed by 2,5-dimethylpyrazine (No. 5, OAV = 31−67), phenylethyl alcohol (No. 16, OAV = 20−38), while 3-ethyl-2,5-dimethylpyrazine (No. 14, OAV = 3−4), benzeneacetaldehyde (No. 12, OAV = 4), 3-butyl-2,5-dimethylpyrazine (No. 19, OAV = 1), and 3-methyl-1-butanol (No. 5, OAV = 1) had the lowest OAVs (all below 4). The results showed that these seven compounds could be represented as the key VOCs to aroma during the submerged fermentation of *T. melanosporum*.

To conclude, seven compounds, i.e., 3-methyl-1-butanol, 2,5-dimethylpyrazine, 3-(methylthio)-1-propanol, benzeneacetaldehyde, 3-ethyl-2,5-dimethylpyrazine, phenylethyl alcohol, and 3-butyl-2,5-dimethylpyrazine, were identified as the key VOCs for aroma by their FD factors combined with their OAVs. In comparing compounds listed in Table 4 (identified by the new strategy) and Table 6 (identified by the traditional method), it is easy to see that all of the VOCs in Table 6 were also contained in Table 4, indicating the reliability of the new strategy and as a viable alternative to the traditional method.

## Discussion

Aroma is an important characteristic for the flavor of fermented food. During the fermentation process of fermented food, microbiology plays a key role in the formation of aroma, and the attributes of aroma are significantly affected by the fermentation conditions^27^,^28^. Aroma consists of the interplay of dozens or even hundreds of VOCs, and only a few key VOCs (also known as aroma active compounds) are the contributors to aroma^19^.

The effects of the fermentation conditions on aroma attributes should be considered. In our previous study, VOC production was most significantly affected by the absence of a major carbon source (i.e., sucrose) and the shift of temperature, followed by the absence of yeast extract and a shift in culture pH^17^. However, the effects of the type of carbon source (i.e., glucose or sucrose) and the levels of these factors (i.e., glucose or sucrose, yeast extract, culture temperature and pH) on VOCs and further effects on aroma production were still unknown. In this work, the optimal fermentation conditions for the aroma attribute of *T. melanosporum* fermentation was 60 g/L glucose and 15 g/L yeast extract maintained at a temperature 28 °C with an initial pH of 7.0 and then without control. Among these fermentation conditions, the nitrogen source (i.e., yeast extract) might be the most important factor for the profile of VOCs due to its richness in amino acids. On the one hand, amino acids also could be utilized for protein formation required for cell growth^29^. On the other hand, amino acids such as L-methionine, isoleucine, leucine, valine and phenylalanine are involved in the Ehrlich pathway to produce many important VOCs for truffles, such as 3-(methylthio)-propanol, 2-methyl-1-butanol, 3-methyl-1-butanol, 2-methyl-1-propanol, and 2-phenylethanol, respectively^30^. It was also reported that variations in amino acid composition account for a high proportion of the variance in the VOCs profile of wine by Hernández-Orte *et al.*^29^.

Generally, to screen and identify the key VOCs for the studies of microbial-producing aroma, the traditional method combining AEDA with OAV, which is based on gas chromatography-olfactometry (GC-O), is employed^20^. However, the traditional method presents a significant challenge for olfactory sense to use GC-O technology for many fermentation samples. First, due to the black-box nature of target aroma attributes, changing the fermentation condition one-at-a-time and repeating the process of AEDA and OAV is time-consuming and laborious. In this study, according to the aroma attributes of *T. melanosporum*, the favorite aroma profile was composed of the following characteristics alcohol, sulfurous^10^, earthy^24^, mushroom^25^, and green^26^. An aroma sensory evaluation method with a total scale point from 0 to 100 (Table 1) was developed to score the fermentation samples obtained from various fermentation conditions and 504 fermentation samples (Table 2) were screened to identify 34 potential favorite aroma samples (scores larger than 50). A disadvantage of AEDA is the length of the total analysis because of the large number of dilutions for each extract and judgment^31^. Additionally, the OAV could be insufficient in identifying the key VOCs for aroma in a sample when the odor intensity increases in parallel with the concentration for all odor components^31^. PCA is a sophisticated technique widely used for reducing the dimensions of multivariate problems. It reduces the dimensionality of the original data set by explaining the correlation among a large number of variables in terms of a smaller number of underlying factors (principle components) without losing much information^32^,^33^. In this study, PCA was employed to screen 9 common key VOCs from these 34 samples, greatly reducing the labor intensity of the olfactory discrimination from fermentation samples. Obviously, in this study, there were 29 VOCs identified in the *T. melanosporum* fermentation system (Table 3), and 26 fermentation conditions of 7 sampling point time (Table 2). Thus, to determine the FD of each VOC, the minimization of dilution samples of 5278 (calculated by 26 × 7 × 29) should be required. Also, to obtain the OAV, it requires to quantitative analysis the 29 VOCs (Table 3) of 26 fermentation conditions of 7 sampling point (from day 1 to 7). Therefore, compared with that of this work, the traditional method offers a challenge for olfactory senses, seems time-consuming and laborious. Finally, as was shown in Table 5, expect the compounds of NO. 16 and NO.20 since both were not considered as the key VOCs for the favorite aroma, seven key VOCs (Table 6) identified by traditional method were also included in the nine key VOCs found by the new strategy, showing that the strategy developed in this work is a viable alternative to the traditional method in studying aroma production.

In summary, the new aroma evaluation method was used to find the favorite fermentation aroma samples, and then PCA was used to screen and identify key volatile organic compound contributors to the favorite aroma. It was demonstrated that the new method shows potential as a viable alternative to the traditional method.

## Materials and Methods

### Chemicals

3-Methyl-1-butanol, 2-methyl-1-butanol, 2,5-dimethylpyrazine, benzeneacetaldehyde, 1-octen-3-ol, 3-octanol, 2-phenylethanol, 3-(methylthio)-1-propanol, and 1-butanol (I.S.) were purchased from Sigma-Aldrich China Inc. (Beijing, China). 3-Ethyl-2, 5-dimethylpyrazine, 3-butyl-2, 5-dimethylpyrazine, trichloroacetic acid tridecyl ester were synthesized and their purity were above 98% in all cases^34^,^35^,^36^. Pure water was obtained using a Milli-Q purification system (Millipore, Bedford, MA, USA).

### Truffle materials

The strain of *T. melanosporum* was kindly provided from Mianyang Institute of Edible Fungi (Building No.110, Hongxing Street, Mianyang City, Sichuan Province, and China). It was maintained on potato-agar-dextrose slants. The slant and preparation of the preculture have been previously described^4^,^5^.

### Sample preparation

The VOCs were extracted by head space solid-phase microextraction (HS-SPME). A volume of 5 mL *T. melanosporum* culture (including mycelia and broth) was directly added into a 15 mL vial closed with one layer of Parafilm-MTM (American National Can, Menasha, WI 54952, USA). The process of HS-SPME was according to one previously described^26^. Specially, the fiber of CAR-PDMS was used, and the headspace exposure time of the sample was 30 min.

### GC-FID and GC-O

A GC-2010 gas chromatograph (GC) equipped with a split/splitless injector and a flame ionization detector (FID) from Shimadzu Technologies Inc. (Tokyo, Japan) was used in this work. The separation was carried out on a HP-5 capillary column (30 m × 0.32 mm i.d., 0.25 μm film thickness) from Agilent Technologies Inc. (CA, USA). The samples were analyzed following a desorption of 2 min at the splitless injection at a split ratio of 1:20 with an injector temperature of 270 °C. The GC oven was programmed such that the temperature started at 40 °C (held for 1 min) increased to 100 °C at a rate of 10 °C min^−1^ (held for 3 min) and then to 130 °C at 5 °C min^−1^ (held for 1 min) and then to 150 °C at 3 °C min^−1^ (held for 1 min), and finally to 250 °C at 15 °C min^−1^ (held for 1 min). Nitrogen (99.999%) was used as the carrier gas at a constant flow rate of 3.0 mL min^−1^. The detector temperature was set to 280 °C.

The GC-O analysis were carried out by using a splitting assembly based on Capillary Flow Technology (Agilent Technologies, USA), where the end of the capillary column is connected, which enables the effluent to be split into the FID and the sniffing port. The split ratio for the olfactometric analysis was 1:1 (FID/sniffing port), and it was achieved by employing two deactivated and uncoated fused silica capillaries of the same width and different length (ODP/FID: 70 cm/155 cm) as a transfer line between the splitting assembly and the detectors. In addition, the ODP2, which incorporates a heated transfer section from the GC oven to the glass detection cone, keeps the unit at a suitable temperature (250 °C) to transfer the VOCs to the detection cone without losses due to condensation. Furthermore, the glass cone is purged with humidified air to prevent nasal mucous membranes from drying out in order to maintain olfactory sensitivity. Sniffers were asked to describe the odor perceived.

### Qualitative analysis of VOCs

The method of qualitative analysis of VOCs was according to our previous report^26^. Specially, volatile compounds were identified by an Agilent 7890A gas chromatography system equipped with a 5975C quadrupole mass spectrometer (MS) detector (CA, USA). Separation was achieved under the same operating conditions described above and using the same column as in the GC-FID and GC-O analyses. The mass spectrometer was operated in the electron impact (EI) mode with electron energy of 70 eV. Interface, source, and quadrupole temperature were 280, 200, and 150 °C, respectively, and the mass range was from 30 to 500 amu. The compounds were identified by the comparison of mass spectra of the target compounds with those of the NIST 08^®^ (National Institute of Standards and Technology) library and verified by the retention time of pure standard compounds.

### Aroma evaluation method

The aroma of *T. melanosporum* fermentation systems were sniffed every day. The tasting panel was composed of seven trained members (four women and three men who aged 24–32) with at least two years of research experience in truffle fermentation. Sensory aroma scores were given according to the aroma evaluation method established in our *T. melanosporum* fermentation systems (Table 1).

### Aroma-producing fermentation conditions optimization

Based on the aroma evaluation method, scores above 50 were considered for the optimization response; the 24 fermentation conditions (carbon source, nitrogen source, culture temperature, and pH), including 168 samples, were ranked by scoring. The details of fermentation conditions are shown in Table 2.

### Data analysis

Data analysis was carried out by using SPSS 19.0 software (SPSS Inc., 233 South Wacker Drive, 11th Floor, Chicago, USA). The Scheffe multiple-range tests (α≤0.05) were employed to assess whether a significant differences existed between the individual variables. The different letters (e.g., a, b, c, d, and e) and their combination (e.g., ab, bc, and abc) in Table 2 were assigned to significantly different groups. For the PCA, data analysis was performed on the common chromatographic peaks in the GC fingerprints. The main chemical markers (i.e., the key VOCs for the favorite aroma fermentation samples), which had the most influence on the favorite aroma among different fermentation samples were identified with the help of PCA loadings plots^32^,^33^.

### Verification test

The Methods of AEDA, the quantitative analysis of the key VOCs and the calculation of OAVs have been previously described^26^. Sniffing of fermentation samples was done by a man (26 years old) and a woman (32 years old) who each had more than three years of research experience in truffle fermentation.

## Additional Information

**How to cite this article**: Liu, R.-S. *et al.* Screening of the key volatile organic compounds of *Tuber* *melanosporum* fermentation by aroma sensory evaluation combination with principle component analysis. *Sci. Rep.* **5**, 17954; doi: 10.1038/srep17954 (2015).

## Figures and Tables

**Table 1:** The sensory evaluation method for *T. melanosporum* fermentation.

Terms	Aroma attribute	Odor descriptor	Scores
The total score (0 – 100) = Basic score+ Additional score			
Basic score (0 ∼ 50) = the Basic line (30) plus the score of the one terms as the follow			
	Spoilage	Spoilage, unpleasant smell	−(20 ∼ 30)
	Acidity	Acidity, sour	−(5 ∼ 19)
	Medium	Medium smell (**Basic line**)	30
	Faint alcohol	Alcohol smell just can be smelled	+(1 ∼ 5)
	Moderate alcohol	Alcohol smell is very obvious	+(6 ∼ 10)
	Strong alcohol	Alcohol smell is very strong	+(11 ∼ 20)
Additional score = the sum of the score of each term as the follow			
	Sulfurous	Sulfurous, boiled potato-like, cabbage	+(0 ∼ 10)
	Mushroom	Mushroom-like	+(0 ∼ 10)
	Earthy	Earthy	+(0 ∼ 10)
	Green	Green	+(0 ∼ 10)
	Others	Fruity, flowery, sweet, hay	+(0 ∼ 10)

**Table 2:** Effects of the carbon source, nitrogen source, culture temperature and pH on the sensory aroma score during the submerged fermentation of *T. melanosporum.*

Terms a	Culture time						
	Day 1	Day 2	Day 3	Day 4	Day 5	Day 6	Day 7
G35Y5	31.0 ± 1.1c	28.8 ± 1.2c	40.2 ± 2.5d	37.1 ± 1.5d	20.7 ± 3.1b	21.0 ± 1.8b	16.3 ± 3.8a
G35Y10	31.8 ± 0.3b	32.9 ± 1.2b	45.6 ± 2.5c	48.3 ± 2.5d	21.1 ± 0.5a	21.3 ± 1.3a	20.3 ± 0.0a
G35Y15	29.0 ± 1.2b	40.0 ± 1.1d	49.3 ± 1.2e	49.6 ± 2.2e	34.5 ± 0.9a	34.4 ± 1.2c	31.1 ± 2.0b
G60Y5	25.5 ± 0.7a	37.8 ± 1.4b	48.0 ± 3.0b	51.5 ± 1.6c	54.7 ± 1.7d	54.4 ± 1.3cd	46.6 ± 2.3b
G60Y10	24.3 ± 0.8a	37.9 ± 2.1b	53.7 ± 3.5c	56.7 ± 2.2cd	58.8 ± 1.1d	59.4 ± 0.8d	57.8 ± 2.9d
G60Y15	32.4 ± 2.2a	48.8 ± 3.6b	55.8 ± 0.2c	58.3 ± 0.9cd	58.5 ± 0.8cd	61.4 ± 0.8d	58.4 ± 1.1cd
G80Y5	33.0 ± 0.9a	34.2 ± 2.4a	47.1 ± 1.2b	49.0 ± 2.7b	52.1 ± 1.8c	54.4 ± 1.5c	53.3 ± 0.6c
G80Y10	24.3 ± 0.5a	35.1 ± 2.2b	47.3 ± 1.1c	56.1 ± 1.4d	57.5 ± 1.3de	59.0 ± 1.0e	56.3 ± 0.9d
G80Y15	31.7 ± 1.1a	42.7 ± 1.9b	49.5 ± 3.0c	58.1 ± 1.1d	58.4 ± 0.6d	57.4 ± 1.9d	56.5 ± 1.2d
S35Y5	29.4 ± 1.5a	39.3 ± 3.6b	40.5 ± 1.2b	37.8 ± 2.8b	27.8 ± 2.8a	38.8 ± 2.6b	39.4 ± 2.4b
S35Y10	21.7 ± 0.5a	28.4 ± 1.4b	37.7 ± 2.0d	37.5 ± 2.2d	23.7 ± 1.8a	33.3 ± 2.6c	32.7 ± 2.8c
S35Y15	22.0 ± 0.7a	35.6 ± 0.7cd	41.3 ± 2.0e	35.9 ± 3.1d	33.3 ± 1.4c	26.7 ± 1.7b	26.8 ± 1.2b
S60Y5	26.5 ± 1.6a	25.2 ± 2.5a	42.5 ± 0.9d	39.6 ± 2.7c	34.7 ± 1.7b	51.1 ± 0.9e	51.0 ± 0.6e
S60Y10	29.0 ± 2.87b	25.4 ± 2.2a	41.0 ± 2.4c	46.3 ± 2.4d	48.2 ± 2.2d	49.1 ± 1.5d	48.8 ± 1.5d
S60Y15	22.7 ± 0.5a	30.9 ± 2.5b	40.6 ± 1.7c	42.5 ± 1.9cd	44.0 ± 3.4cd	44.7 ± 2.9d	44.3 ± 2.9cd
S80Y5	24.8 ± 1.4b	35.5 ± 1.8c	39.5 ± 2.8d	21.3 ± 0.6a	40.7 ± 2.8d	45.6 ± 2.0e	43.7 ± 1.0e
S80Y10	22.4 ± 0.5a	39.5 ± 1.3b	37.6 ± 0.8b	52.2 ± 1.4d	49.2 ± 2.6c	50.5 ± 2.9cd	52.5 ± 1.5d
S80Y15	24.5±1.4a	28.9 ± 1.8b	43.4 ± 1.2e	38.9 ± 2.6c	42.2 ± 3.0de	33.0 ± 2.2c	30.0 ± 1.3b
T^c^25 °C	32.4 ± 2.2a	48.8 ± 3.6b	55.8 ± 0.2c	58.3 ± 0.9cd	58.5 ± 0.8cd	61.4 ± 0.8d	58.4 ± 1.1cd
T28 °C	30.0 ± 0.0a	35.0 ± 0.0a	41.3 ± 7.4b	57.5 ± 4.3c	61.3 ± 2.2c	71.6 ± 2.1d	69.8 ± 6.3d
T30 °C	17.8 ± 1.8a	28.3 ± 2.0b	30.0 ± 7.1b	52.5 ± 2.5c	52.5 ± 7.5c	55.0 ± 9.4c	31.3 ± 7.4b
T32 °C	20.0 ± 3.1b	12.5 ± 1.8ab	19.5 ±7.5b	17.5 ± 4.3ab	17.5 ± 8.3ab	12.5 ± 8.3ab	7.5 ± 4.3a
I^d^7.0	30.0 ± 0.0a	35.0 ± 0.0a	41.3 ± 7.4b	57.5 ± 4.3c	61.3 ± 2.2c	71.6 ± 2.1d	69.8 ± 6.3d
pH^d^6.0	30.0 ± 0.0ab	39.2 ± 5.8bcd	32.0 ± 13.5a	35.0 ± 10.6abc	33.8 ± 8.5ab	45.4 ± 9.5cd	50.0 ± 11.7d
pH5.5	30.0 ± 0.0b	30.0 ± 5.5b	35.0 ± 7.9c	36.0 ± 2.2c	40.0 ± 4.1c	36.0 ± 8.9c	20.6 ± 2.6a
pH4.0	40.0 ± 0.0ab	30.8 ± 5.8a	38.0 ± 2.7ab	40.6 ± 9.0bc	54.7 ± 5.3c	63.6 ± 9.3d	65.0 ± 12.2d

^a^G35 indicates 35 g L^−1^ of glucose, Y5 indicates 5 g L^−1^ of yeast extract, S35 indicates 35 g L^−1^ of sucrose. Other medium compositions: Peptone 5 g/L, MgSO_4_.7H_2_O 0.5 g/L, KH_2_PO_4_ 1 g/L and vitamin B_1_ 0.05 g/L. Culture condition: rotary shaker at a speed of 120 rpm under natural light with an initial pH of 7.0 without controlled and culture temperature of 25 °C.

^b^The sensory score, mean ± S.D. (n = 5).

^c^The culture temperature was maintained at 25 °C, 28 °C, 30 °C and 32 °C. Other culture conditions: rotary shaker at a speed of 120 rpm under natural light with an initial pH of 7.0 without further control. The medium composition: glucose 60 g/L, yeast extract 15 g/L, peptone 5 g/L, MgSO_4_.7H_2_O 0.5 g/L, KH_2_PO_4_ 1 g/L and vitamin B_1_ 0.05 g/L.

^d^I7.0 indicates initial pH 7.0 without further control, and pH 6.0, pH 5.5, and pH 4.0 indicates the culture pH was maintained at 6.0, 5.5, 4.0, respectively. Other culture conditions: rotary shaker at a speed of 120 rpm under natural light with a culture temperature of 28 °C. The medium composition: glucose 60 g/L, yeast extract 15 g/L, peptone 5 g/L, MgSO_4_.7H_2_O 0.5 g/L, KH_2_PO_4_ 1 g/L and vitamin B_1_ 0.05 g/L.

**Table 3:** Identified compounds and their retention time in the *T*. *melanosporum* fermentation system.

NO.^a^	LRI	Compound	Mode of identification	S.I (%)
1	<600	Ethanol	MS	86
2	<600	2-Methyl-1-propanol	MS, std	93
3	658	3-Methyl-1-butanol	MS, std	90
4	800	2, 3-Butanediol	MS	83
5	851	2, 5-Dimethylpyrazine	MS, std	90
6	911	Benzaldehyde	MS, std	79
7	914	1-Octen-3-ol	MS, std	86
8	929	3-Octanol	MS, std	84
9	931	3-(Methylthio)-1-propanol	MS, std	93
10	938	Trimethylpyrazine	MS, std	81
11	962	Cycloocta-2, 7-dienone	MS	79
12	982	Benzeneacetaldehyde	MS, std	70
13	1001	1-Octanol	MS	83
14	1007	3-Ethyl-2, 5-dimethylpyrazine	MS, std	93
15	1047	Phenylethyl alcohol	MS	94
16	1104	4-Ethylphenol	MS	93
17	1110	2-Ethylphenol	MS	81
18	1164	(E)-9-Eicosene	MS	78
19	1183	3-Butyl-2, 5-dimethylpyrazine	MS, std	81
20	1184	2, 5-Dimethyl-3-propylpyrazine	MS, std	77
21	1237	Octamethylcyclotetrasiloxane	MS	83
22	1239	Trichloroacetic acid tridecyl ester	MS	78
23	1245	2, 3, 4-Trimethyl-1, 4-pentadiene	MS	78
24	1262	1-Methylcycloundecene	MS	70
25	1305	1, 2, 3, 4-Tetrahydro-1, 1-dimethylnaphthalene	MS	70
26	1388	Decamethyl cyclopentasiloxane	MS	79
27	1391	1, Z-5, E-7-Dodecatriene	MS	70
28	1554	1, 1′-(1, 3-Propanediyl)bis-benzene	MS	88
29	2034	Hexanoic-acid-2-ethyl-oxybis ester	MS	72

^a^The ‘No.’ of compounds from 1 to 29 was according to the linear retention index (LRI) from low to high, and each identified compound was reported with their matching index (S.I) to the NIST‘05 database (MS). Volatiles were identified by one or more methods mentioned under column ‘Mode of identification’ as ‘MS’ (comparing their MS data to the NIST‘05 database), and ‘std’ (using authentic standards under similar GC–MS conditions for comparison).

**Table 4:** The 11 key VOCs for 34 fermentation samples with sensory aroma score larger than 50.

No.	Compounds^a^	Component 1 (50.4%)^b^	Component 2 (16.9%)	Component 3 (13.4%)
3	3-Methyl-1-butanol	−0.03	−0.31	−0.80
4	2,3-Butanediol	0.84	0.31	−0.24
5	2,5-Dimethylpyrazine	0.39	−0.68	0.08
9	3-(Methylthio)-1-propanol	0.74	0.37	−0.24
10	Trimethylpyrazine	0.38	−0.77	0.01
12	Benzeneacetaldehyde	0.75	0.14	−0.26
14	3-Ethyl-2,5-dimethylpyrazine	−0.03	0.33	0.76
15	Phenylethyl alcohol	−0.07	0.18	0.65
16	4-Ethylphenol	0.73	0.31	−0.05
19	3-Butyl-2,5-dimethylpyrazine	0.10	−0.21	0.73
20	2,5-Dimethyl-3-propylpyrazine	0.75	-0.05	0.17

^a^The compounds and its number were consistent with the Table 3.

^b^The contributing of the component to the VOCs for the 34 fermentation samples.

**Table 5:** The effect of high sensory scores (larger than 50), obtained under the nine fermentation conditions, on the average relative peak are of the key VOCs.

No.	Compound^a^	The samples with its sensory aroma scores^b^									
		G80Y5	G80Y10	G80Y15	G60Y5	G60Y10	G60Y15	T30	pH4.0	Control	The average relative peak area (%)^d^
		53.27 ± 1.15	57.23 ± 1.34	57.60 ± 0.84	53.53 ± 1.44	57.28 ± 2.01	58.48 ± 1.98	53.33 ± 1.44	62.17 ± 6.97	65.05 ± 6.75	
		The average relative peak area of the compound (%)^c^									
3	3-Methyl-1-butanol	13.55	14.41	49.95	29.86	52.42	29.00	23.49	30.52	31.00	30.47 ± 13.49
4	2,3-Butanediol	0.24	0.11	0.15	0.17	0.17	0.49	0.50	1.46	0.44	0.41 ± 0.42
5	2,5-Dimethylpyrazine	0.53	0.05	1.10	0.34	0.18	1.39	0.70	0.42	1.41	0.68 ± 0.51
9	3-(Methylthio)-1-propanol	0.16	0.16	0.08	0.20	0.02	0.09	0.17	1.90	0.09	0.32 ± 0.59
10	Trimethylpyrazine	0.22	0.07	0.54	0.17	0.05	0.42	0.14	0.19	0.38	0.24 ± 0.17
12	Benzeneacetaldehyde	0.18	0.05	0.17	0.03	0.04	0.15	0.21	1.23	0.13	0.24 ± 0.37
14	3-Ethyl-2, 5-dimethylpyrazine	1.28	0.80	0.25	0.17	0.12	0.32	0.33	0.24	0.31	0.42 ± 0.38
15	Phenylethyl alcohol	51.20	65.29	28.44	50.66	29.52	44.47	57.97	42.39	43.29	45.91 ± 12.08
16	4-Ethylphenol*	0.08	0.04	0.00	0.01	0.00	0.38	0.01	0.47	0.39	0.15 ± 0.20
19	3-Butyl-2, 5-dimethylpyrazine	0.44	0.43	0.05	0.12	0.13	0.27	0.23	0.13	0.30	0.23 ± 0.14
20	2,5-Dimethyl-3-propylpyrazine	0.06	0.02	0.03	0.02	0.02	0.06	0.00	0.04	0.07	0.04 ± 0.02

^a^The compounds and their numbers were consistent with the Table 3.

^b^A sensory score was the mean ± SD calculated from the samples with the sensory scores larger than 50 obtained under a particular fermentation condition. The G80Y5, G80Y10, G80Y15, G60Y5, G60Y10, G60Y15 conditions are the same as in the Table 1. T30: culture temperature of 30 °C, pH4.0: the culture pH was maintained at 4.0. The control uses the optimal aroma-producing fermentation conditions.

^c^The average relative peak area was calculated from the compound in the samples with sensory aroma scores larger than 50 obtained under a particular fermentation condition.

^d^The average relative peak area was calculated from the same row of the compound’s relative peak area.

^*^The compounds were not identified as the key VOCs for the favorite aroma.

**Table 6:** Gas chromatographic retention data, olfactory description, and chemical identity, flavor dilution factor and odor active values for each compound in the fermentation samples obtained under the optimal aroma-producing fermentation conditions in this study.

LRI^a^	Compound^b^	Odor threshold in water (μg/L)	Odor descriptor^e^	Flavor dilution factor (Odor active values)				
				Day 2	Day 4	Day 5	Day 6	Day 7
658	3-Methyl-1-butanol(NO. 3)	300.0^c^		−f	8 ± 0	4 ± 0	4 ± 0	2 ± 0
		Liu *et al.* (2012)	Cheese	(−)^g^	(1 ± 0.08)	(1 ± 0.20)	(1 ± 0.07)	(1 ± 0.14)
851	2,5-Dimethylpyrazine(NO. 5)	1.8		—	2 ± 0	4 ± 0	32 ± 0	32 ± 0
		Guadagni *et al.* (1972)^d^	Nutty, raw-potato	(−)	(55 ± 0.80)	(35 ± 0.91)	(67 ± 0.73)	(31 ± 0.81)
931	3-(Methylthio)-1-propanol(NO. 9)	1.2		—	—	—	—	1 ± 0
		Mestres *et al.* (2000)	Potato, soup, meat like	(−)	(−)	(−)	(−)	(117 ± 10.09)
982	Benzeneacetaldehyde(NO. 12)	1.0	Styrene, daisy	—	—	—	1	—
		Liu *et al.* (2012)		(−)	(−)	(−)	(4 ± 0.22)	(−)
1007	3-Ethyl-2, 5-dimethylpyrazine(NO. 14)	8.6	Nutty, roasted-potato	4 ± 0	8 ± 0	2 ± 0	—	—
		Buttery (1999)		(3 ± 0.13)	(4 ± 0.31)	(4 ± 0.27)	(−)	(−)
1047	Phenylethyl alcohol(NO. 15)	1100.0		2 ± 0	2 ± 0	1 ± 0	4 ± 0	—
		Liu *et al.* (2012)	Rose flower	(20 ± 0.41)	(38 ± 0.78)	(27 ± 1.53)	(26 ± 1.02)	(−)
1183	3-Butyl-2, 5-dimethylpyrazine(NO. 19)	8.0		16 ± 0	4 ± 0	2 ± 0	8 ± 0	1 ± 0
		Buttery (1999)	Green	(1 ± 0.02)	(1 ± 0.11)	(1 ± 0.03)	(1 ± 0.04)	(3 ± 0.18)

^a^The linear retention index (LRI).

^b^Identification based on comparison between gas chromatographic retention indices and mass spectrometric data with those of the pure compounds available in the lab. The No. was consistent with Table 3.

^c^The odor threshold in water of the corresponding compound (μg/L).

^d^Source of the odor threshold of reference compounds.

^e^Odor quality perceived through the sniffing port.

^f^Flavor dilution factor of the corresponding compound. The mean ± SD was calculated from the two judges.

^g^The OAV was calculated by the ratio of the absolute concentration to the odor threshold of each compound. The mean ± SD was calculated from the three samples.
